# Paternal Allele Influences High Fat Diet-Induced Obesity

**DOI:** 10.1371/journal.pone.0085477

**Published:** 2014-01-08

**Authors:** Sumiyo Morita, Takuro Horii, Mika Kimura, Yuji Arai, Yasutomi Kamei, Yoshihiro Ogawa, Izuho Hatada

**Affiliations:** 1 Laboratory of Genome Science, Biosignal Genome Resource Center, Institute for Molecular and Cellular Regulation, Gunma University, Showa-machi Maebashi, Japan; 2 Division of Developmental Biotechnology, Department of Bioscience and Genetics, Research Institute, National Cerebral and Cardiovascular Center, Fujishiro-dai, Suita, Osaka, Japan; 3 Laboratory of Molecular Nutrition, Graduate School of Environmental and Life Science, Kyoto Prefectural University, Hangi-cho, Shimogamo, Sakyo-ku, Kyoto, Japan; 4 Department of Molecular Endocrinology and Metabolism, Graduate School of Medical and Dental Sciences, Tokyo Medical and Dental University, Bunkyo-ku, Yushima, Tokyo, Japan; University of Cordoba, Spain

## Abstract

C57BL/6J (B6) mice are susceptible to high-fat diet (HFD)-induced obesity and have been used in metabolism research for many decades. However, the genetic component of HFD-induced obesity has not yet been elucidated. This study reports evidence for a paternal transmission of HFD-induced obesity and a correlated expression of *Igf2* and *Peg3* (paternal expressed gene 3) imprinted genes. We found that PWK mice are resistant to HFD-induced obesity compared to C57BL/6J mice. Therefore, we generated and analyzed reciprocal crosses between these mice, namely; (PWK×B6) F1 progeny with B6 father and (B6×PWK) F1 progeny with PWK father. The (PWK×B6) F1 mice were more sensitive to diet-induced obesity compared to (B6×PWK) F1 mice, suggesting a paternal transmission of diet-induced obesity. Expression analysis of imprinted genes in adipocytes revealed that HFD influences the expression of some of the imprinted genes in adipose tissue in B6 and PWK mice. Interestingly, *Igf2* and *Peg3*, which are paternally expressed imprinted genes involved in the regulation of body fat accumulation, were down-regulated in B6 and (PWK×B6) F1 mice, which are susceptible to HFD-induced obesity, but not in PWK and (B6×PWK) F1 mice, which are resistant. Furthermore, in vitro analysis showed that *Igf2*, but not *Peg3*, had an anti-inflammatory effect on TNF-α induced MCP-1 expression in adipocytes. Taken together, our findings suggest that the down-regulation of *Igf2* and *Peg3* imprinted genes in adipocytes may be involved in the paternal transmission of HFD-induced obesity.

## Introduction

In mammals, the maternal and paternal genomes are not functionally equivalent. This is due to a small number of genes called imprinted genes, which exhibit the parental allele-specific expression. There are two types of imprinted genes, namely, paternally expressed genes, in which the paternal allele is expressed and the maternal allele is repressed, and maternally expressed genes, in which the maternal allele is expressed and the paternal allele is repressed.

Several imprinted genes are associated with body weight dysregulation. In humans, genetic disorders affecting imprinted genes lead to obesity. Prader-Willi syndrome (PWS) is a genetic disorder resulting from the loss of expression of a cluster of paternally expressed genes on chromosome 15q11-q13 [1], which leads to obesity. *Peg1/Mest* is a paternally-expressed gene1/mesoderm-specific transcript, which promotes adipogenesis and obesity when overexpressed [2], [3]. *Pref1/Dlk1* (preadipocyte factor 1/Delta, Drosophila Homolog-like 1) is another paternally expressed gene that is expressed in preadipocytes and inhibits the differentiation of preadipocytes into mature adipocyte [4]. In addition, *Pref1/Dlk1*-null mice develop adult-onset obesity [5], while mice overexpressing *Pref1/Dlk*1 in adipose tissue develop a lean phenotype with glucose intolerance [6]. Moreover, *Peg3* knockout mice are characterized by increased body fat [7]. Other imprinted genes, such as *Igf2*, *Igf2r*, and *p57Kip2*, may be involved in the regulation of adipocyte growth [8].

Obesity, which is defined as an increase in white adipose tissue (WAT) mass, arises from a complex interaction between genetic and environmental factors. Over the last 30 years, the incidence of obesity has increased rapidly in industrial countries, and has led to an increase in obesity associated metabolic disease, such as type 2 diabetes, and cardiovascular disease. It is therefore crucial that we gain greater understanding of the molecular and genetic mechanisms governing the development of obesity.

The C57BL/6J mouse strain is susceptible to HFD-induced obesity and type 2 diabetes [9], and has been used in metabolism research for many decades. There are several studies that have characterized inbred mouse strains with a differential tendency to obesity [10]. C57BL/6J, AKR/J, and DBA/2J mice are susceptible to diet-induced obesity, while A/J, KsJ, and SWR/J mice are resistant to obesity. In the present study, we compared the tendency to obesity between C57BL/6J and the wild-derived strain, PWK mice, and found that PWK mice are resistant to obesity. In addition, we generated and analyzed reciprocal crosses between these mice, (PWK×B6) F1 and (B6×PWK) F1 mice. The (PWK×B6) F1 mice were more sensitive to dietary obesity compared to (B6×PWK) F1 mice. These results suggested a paternal transmission of diet-induced obesity.

We analyzed several of the imprinted genes involved in obesity, in adipocytes of mice fed a control diet and mice fed a HFD, and found that the paternal transmission of obesity correlated with the expression of the imprinted genes, *Igf2* and *Peg3*, which might contribute to the symptoms associated with obesity.

## Materials and Methods

### Ethics Statement

All animal experiments were approved by the Animal Care and Experimentation Committee, Gunma University, Showa Campus, Japan, and were conducted according to the guidelines of this committee.

### Animals and Diets

C57BL/6J mice were purchased from Charles River Japan. PWK mice were purchased from RIKEN BioResource Center, Tsukuba, Japan. Mice were housed in box cages, maintained on a 12-h light/12-h dark cycle. C57BL/6J and PWK male mice and F1 male progeny obtained by reciprocal crosses between them were placed into single cages and fed for 15 weeks either a normal diet (Research Diets, New Brunswick, NJ, D06072701) or a HFD (Research Diets, New Brunswick, NJ, D07012601) from 6-weeks-old.

### Glucose Tolerance Test

A glucose tolerance test was carried out on animals that had been fasted for 16 h. After determination of fasted glucose levels, each animal received an intraperitoneal (i.p.) injection of 2 g/kg body weight glucose. Blood glucose levels were detected after 30, 60, 90, 120, and 180 min. Areas under the curves (AUCs) for plasma glucose levels (AUCglucose) were calculated using the trapezoidal rule.

### Cell Culture

The 3T3-L1 cells were purchased from American Type Culture Collection (Manassas, VA). Cells were grown and maintained in DMEM containing 10% fetal bovine serum. For adipocyte differentiation, cells were treated with standard growth medium described above supplemented with 1.7 M insulin (Sigma), 0.5 uM dexamethasone (Sigma), and 0.8 mM isoobutylmethylxanthine (IBMX, Sigma) for 3 days. After 3 days, the medium was replaced with growth medium supplemented with 1.7 M insulin only. Medium was changed every 2 days. For chronic treatment with TNF-α (3 ng/ml of TNF-α for 72 h), differentiated 3T3-L1 adipocytes (day 7) were starved overnight in 0.5% serum containing media prior to stimulation with TNF-α, and media was replaced with fresh media every 24 h. For down-regulation of *Peg3* in differentiated 3T3-L1, we transfected siRNA of *Peg3* (Gene Solution siRNA, Qiagen) into differentiated 3T3-L1 (day 4) using Lipofectoamine 2000 (Invitrogen). After 2 days of transfection, cells were collected and subjected to total RNA preparation using Trizol (Invitrogen). To overexpress *Peg3* in 3T3-L1 cells, mouse *Peg3* cDNA was inserted into the pcDNA™ 3.1 Directional TOPO Expression vector (Invitrogen) using PCR with primers designed to amplify the open reading frame of *Peg3*. Cells were transfected using Lipofectamine 2000 (Invitrogen). As a control, the expression vector without *Peg3* cDNA was transfected. Stably transfected cells were selected using G418 (800 mg/mL) prior to experimental analysis.

### 2-Deoxyglucose uptake Assay

Differentiated 3T3-L1 adipocytes were starved in low-glucose DMEM with 0.5% serum overnight prior to insulin stimulation. 2-Deoxyglucose uptake assays were performed with 2-Deoxyglucose (2DG) Uptake Measurement Kit (Cosmo Bio, Japan).

### Quantitative RT-PCR Analysis

Total RNA was prepared from isolated tissues using the Allprep DNA/RNA mini kit (Qiagen). Gene expression levels were measured with LightCycler 480 (Roche) using the SYBR Premix Ex Taq (TAKARA) according to the manufacturer’s instructions. Expression levels were normalized against the level of 18S ribosomal RNA. Primer sequences were as follows:

Igf2: 5′-TGACACGCTTCAGTTTGTCTG-3′, 5′-AAGCAGCACTCTTCCACGAT-3′

Peg1: 5′-GGCCTACGCATCTTCTACCA-3′, 5′-TGGATGTTGGAAAGCCATGT-3′,

Peg3: 5′-AGCCGAAGTGGGAGAGAAAC-3′, 5′-TCTCGAGGCTCCACATCTCT-3′

Dlk1: 5′-TGTGACCCCCAGTATGGATT-3′, 5′-CCAGGGGCAGTTACACACTT-3′

Zac1: 5′-AGTGCTCGAAGGCTGAGTGT-3′, 5′-TCTGGAGGTGGTTCTTCAGG-3′

Grb10: 5′-CAGGACTCAGCATTGGTTCC-3′, 5′-CAGTACGAACGCCTTTGGAT-3′

MCP1: 5′-AGCACCAGCCAACTCTCACT-3′, 5′-CGTTAACTGCATCTGGCTGA-3′

Glut4:5′-CTGTCGCTGGTTTCTCCAAC-3′, 5′-CAGGAGGACGGCAAATAGAA-3′

Adiponectin: 5′-GATGGCACTCCTGGAGAGAA-3′, 5′-GCTTCTCCAGGCTCTCCTTT-3′

18s: 5′-CCCGAAGCGTTTACTTTGAA-3′, 5′-CCCTCTTAATCATGGCCTCA-3′.

### Bisulfite DNA Methylation Analysis

Genomic DNA of isolated tissues was prepared using the Allprep DNA/RNA mini kit (Qiagen), and treated with the Epitect Plus DNA Bisulfite Kit (Qiagen) according to the manufacturer’s instruction. The modified DNA was amplified with the primer as follows:

Peg3: 5′-ATTTTGTTTGGGGGTTTTTAATATT-3′, 5′-CCTATCACCTAAATAACATCCCTAC-3′.

Igf2: DMR1-F 5′-TTTTTATTTTTGGTTTTTTTGGTTTT-3′, DMR1-R 5′-ACTACCCTCTCAAATACCCCTTAAA-3′, DMR2-F1 5′-TTGGAGAGGAGGTGTTAGTTAGTAG-3′, DMR2-R1 5′-AAAAATAATTCCTTCTTCAACCTTC-3′ DMR2-F2 5′-TTTATTGATGGTTGTTGGATATTTT-3′, DMR2-R2 5′-CTAACTAACACCTCCTCTCCAAAAC-3′ H19-DMR-F 5′-TATAGGGGTGGTAAGATGTGTGTATT-3′, H19-DMR-R 5′-CTTAAAAAATCCCAAAACAAAAAAA-3′.

The amplified fragments were ligated into the TOPO vector (Invitrogen) and sequenced.

### Statistical Analysis

Student’s t-test was used for single comparisons and ANOVA was used for multiple comparisons.

## Results

### The Paternal Allele Influences Body Weight Gain

**Figure 1:**
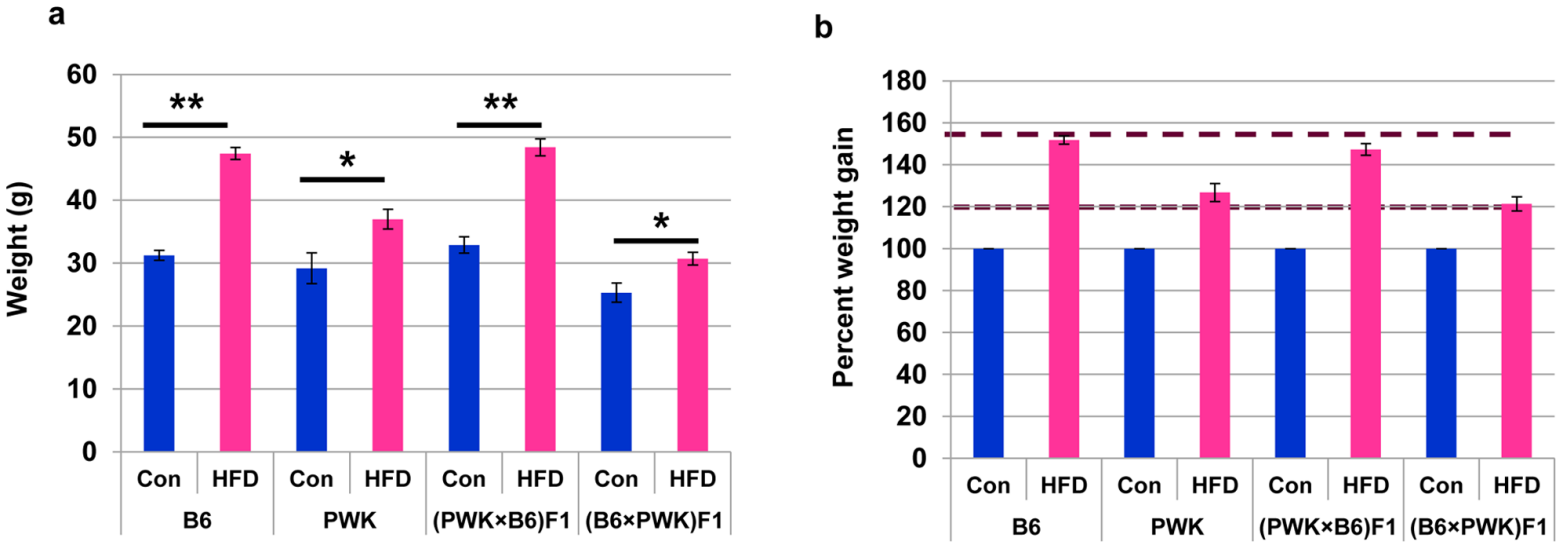
Body weight changes in B6, PWK, and the progeny of their reciprocal crosses. (a) Body weight of B6, PWK, (PWK×B6) F1 and (B6×PWK) F1 mice fed normal chow and HFD for 15w. (b) Comparison of body weight normalized to mice fed normal chow. *P≤0.05, **P≤0.01.

C57BL/6J (B6) mice, PWK mice and the F1 progeny obtained by reciprocal crosses between them, at the age of 6 weeks, were fed, for 15 weeks, either a normal control diet or a HFD. The body weight of B6 mice fed the HFD was approximately 50% higher compared to B6 mice fed the control diet, while PWK mice fed the HFD was only 20% heavier compared to PWK mice fed the control diet (Fig. 1a, b), suggesting that B6 mice are more susceptible to obesity than PWK mice. HFD also resulted in approximately 50% higher body weight gain in (PWK×B6) F1 mice, compared to the same mice fed the control diet (Fig. 1a, b). By contrast, (B6×PWK) F1 mice fed the HFD had only 20% heavier body weight gain compared to the same mice fed the control diet. Thus, F1 mice with B6 father gain more weight than those having PWK father, suggesting that paternally inherited factors influence the HFD-induced obesity in B6 mice.

### Glucose Tolerance is Linked with the Paternal Allele

**Figure 2:**
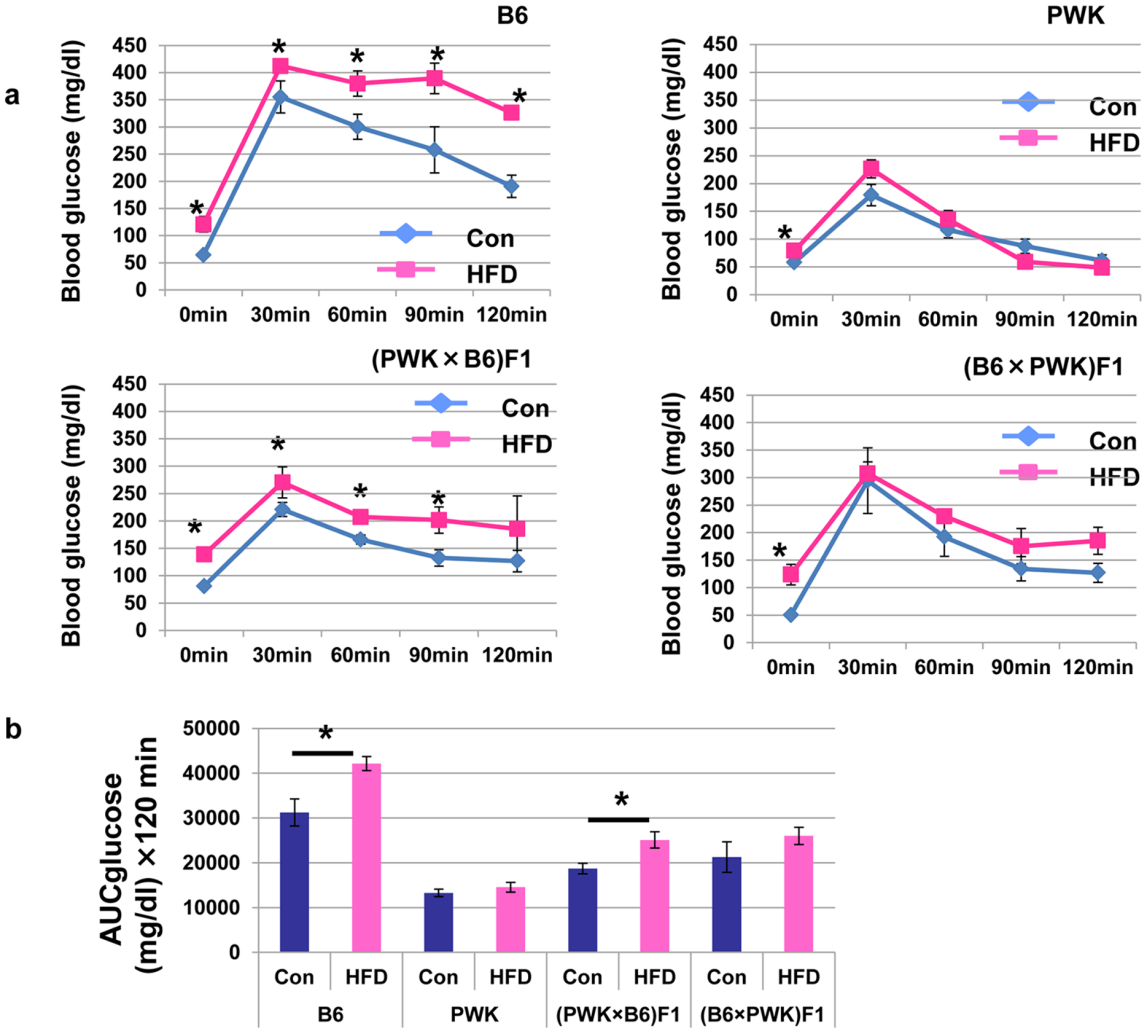
Effect of diet on glucose tolerance in B6, PWK, (PWK×B6) F1 and (B6×PWK) F1. (a) Glucose tolerance tests were performed after an overnight fast in B6, PWK, (PWK×B6) F1 and (B6×PWK) F1 fed normal chow and HFD. *P≤0.05. (b) AUC-glucose represents the total clearance of plasma glucose between 0 min and 120 min. *P≤0.05.

B6 mice fed a HFD showed impaired glucose tolerance compared to those fed a control diet (Fig. 2a). By contrast, PWK mice fed a HFD showed normal glucose tolerance similar to mice fed a control diet. (PWK×B6) F1 mice fed a HFD showed impaired glucose tolerance compared to the same mice fed a control diet, while (B6×PWK) F1 mice fed a HFD showed a normal glucose tolerance. This was also illustrated by measurement of AUCglucose (Fig. 2b). These data suggest that impaired glucose tolerance induced with a HFD is dependent on the paternal allele, the same as for obesity.

### High-fat Diet Alters the Expression of Imprinted Genes in WAT

**Figure 3:**
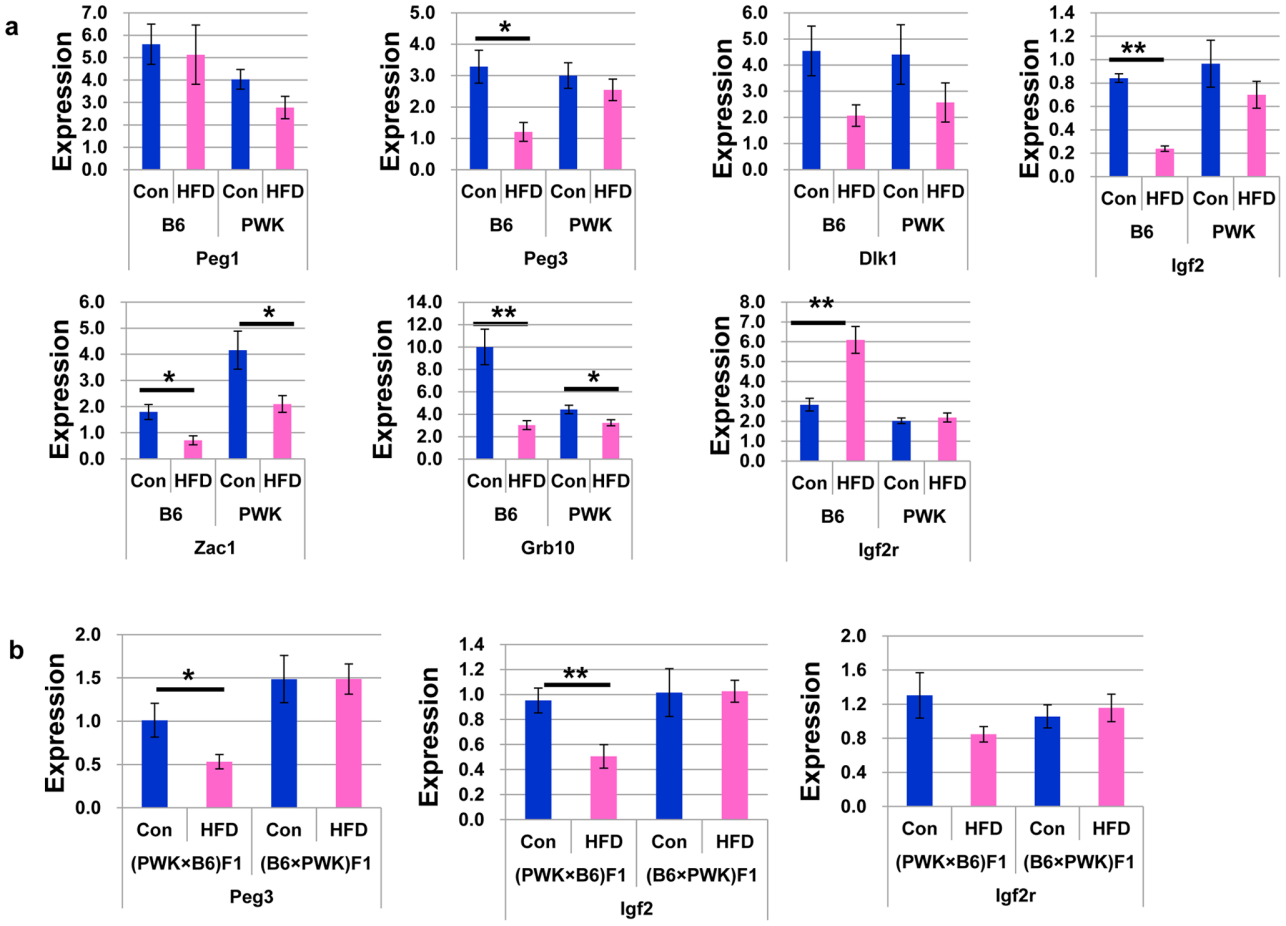
Expression of imprinted genes in adipose tissue. (a) Expression of imprinted genes in adipose tissue of B6 and PWK mice fed control diet and HFD. *P≤0.05, **P≤0.01. (b) Expression of *Peg3*, *Igf2*, and *Igf2r* in adipose tissue of (PWK×B6) F1 and (B6×PWK) F1 mice fed control diet and HFD. *P≤0.05, **P≤0.01.

Several imprinted genes have been associated with the development of obesity through effects on adipocyte differentiation and function. Therefore, we analyzed the expression of seven imprinted genes, which could be involved in adipocyte differentiation and insulin signaling, in WAT of the parental strains B6 and PWK (Fig. 3a). In B6 mice, *Igf2r* expression was increased in WAT of diet-induced obese mice. By contrast, the expression of *Peg3*, *Igf2*, *Zac1 and Grb10* was significantly decreased in WAT of diet-induced obese B6 mice. In PWK mice, *Igf2r* expression was unchanged in WAT of diet-induced obese mice and control mice, and *Zac1* and *Grb10* expressions was decreased in WAT of diet-induced obese mice compared to control mice, similar to B6 mice, although the expression of *Peg3* and *Igf2* was not affected in WAT of obese PWK mice. Therefore, the down-regulation of *Peg3* and *Igf2* and up-regulation of *Igf2r* in adipocytes from HFD-induced obesity mice is specific to B6 mice. Changes in the expression of other imprinted genes were observed in both B6 and PWK mice.

To clarify whether the down-regulation of these paternally expressed imprinted genes is dependent on the B6 allele, we further analyzed the expression of *Peg3* and *Igf2*, which are paternally expressed genes, and *Igf2r*, which is maternally expressed gene, in (PWK×B6) F1 mice and (B6×PWK) F1 mice (Fig. 3b). In (PWK×B6) F1 mice, HFD down-regulates the expression of *Peg3* and *Igf2*, while in (B6×PWK) F1 mice, HFD does not alter the expression of *Peg3* and *Igf2*. However, *Igf2r* expression was not altered in both (PWK×B6) F1 and (B6×PWK) F1 mice. Therefore, the down-regulation of the paternally expressed genes, *Peg3* and *Igf2*, occurs only in the F1 offspring with B6 father.

### High Fat Diets Induce Down-regulation of Peg3 and Igf2 in WAT, which is not Caused by Alteration of DNA Methylation Status in the Differentially Methylated Region (DMR)

**Figure 4:**
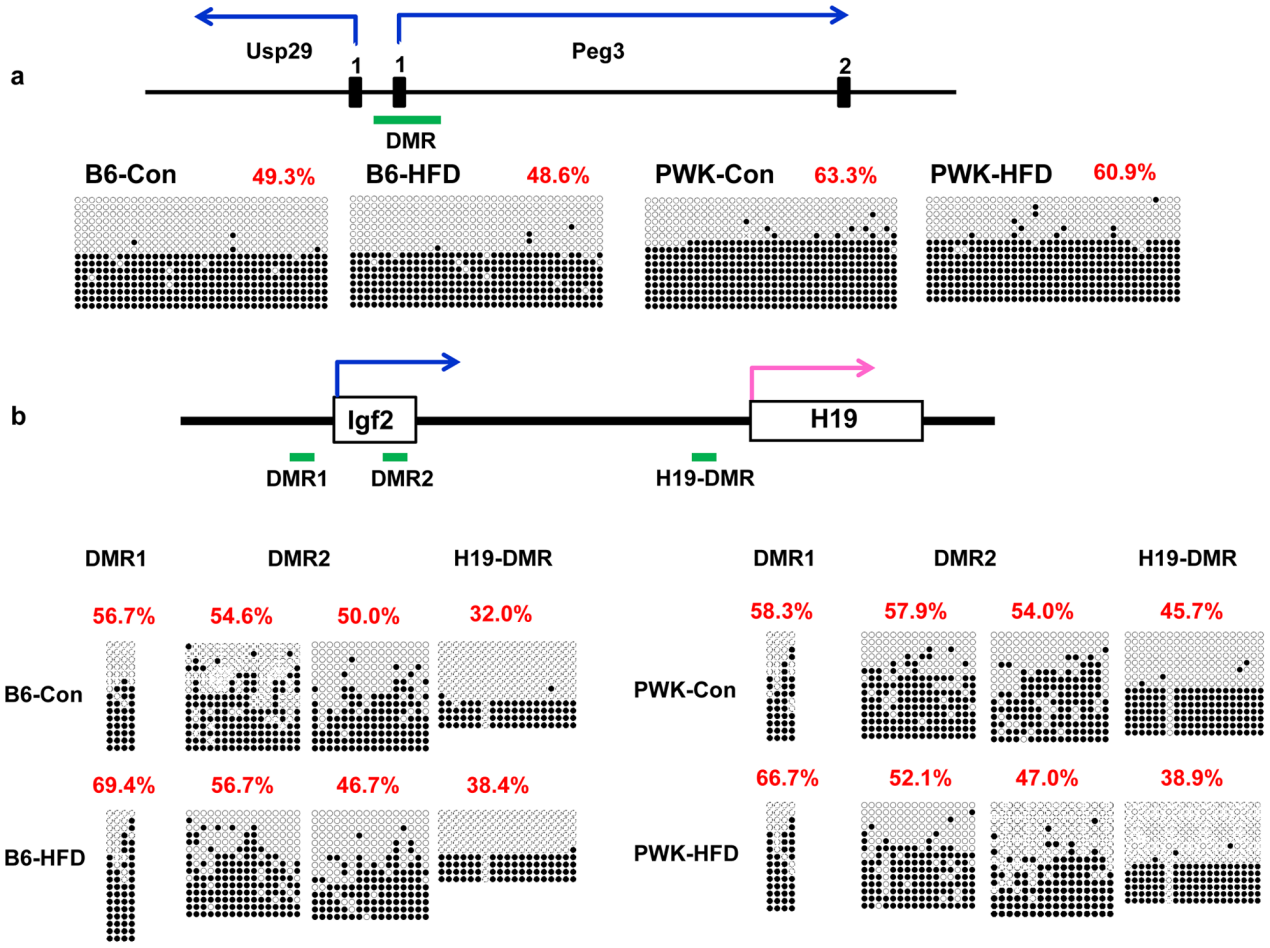
The DNA methylation status of the *Peg3* and *Igf2* DMR. Analysis of *Peg3* (a) and *Igf2* (b) DMR were performed by bisulfite sequencing in B6 and PWK mice fed with control diet and HFD. Methylated and unmethylated CpG sites are indicated by closed and open circles, respectively. Percentages of methylated CpG sites are indicated in parentheses.

*Peg3* and *Igf2* were down-regulated in WAT of B6 obese mice, and therefore we examined the DNA methylation status of the *Peg3* and *Igf2* DMR [11], [12], [13] in WAT of B6 mice fed a control diet and mice fed a HFD, using the bisulfite sequencing method. As shown in Fig. 4 (a, b), there was no significant difference in the DNA methylation status in the DMR of *Peg3* and *Igf2*, suggesting that *Peg3* and *Igf2* down-regulation in WAT of B6 obese mice is not caused by alterations of DNA methylation in their DMRs. We also examined the DNA methylation statuses of the *Peg3* and *Igf2* DMRs in WAT of PWK mice fed a control diet or a HFD. As expected, there was also no significant difference in the DNA methylation status of the DMRs of *Peg3* or *Igf2* (Fig. 4a, b).

### Igf2 Protects Adipocytes from the Inflammatory Effect of TNF-α

**Figure 5:**
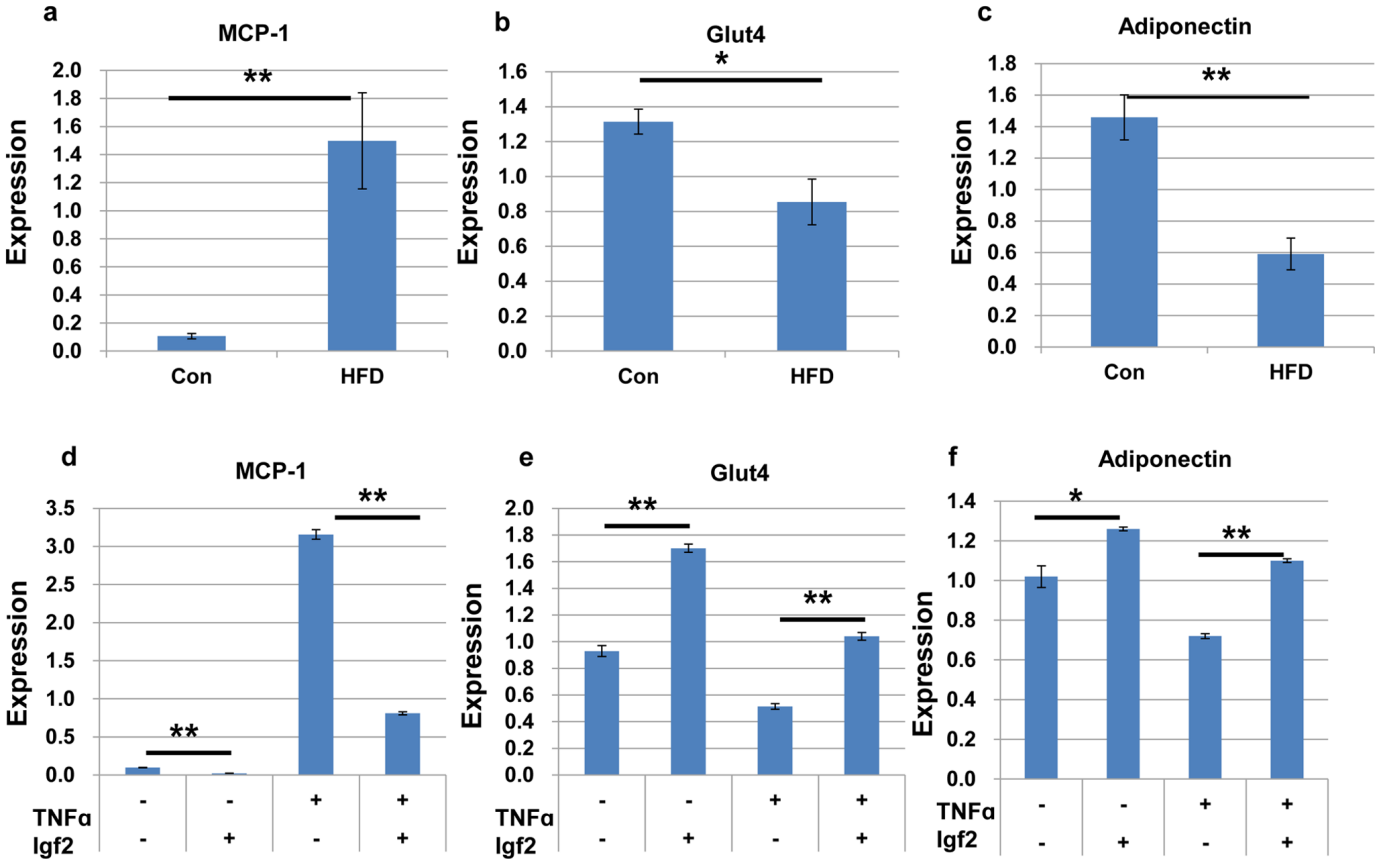
The effect of Igf2 on the expression of *MCP-1*, *Glut4* and *adiponectin*. Expression of *MCP-1* (a,*Glut4* (b), and *adiponectin* (c) in WAT of B6 mice fed a control diet or a HFD. Expression of *MCP-1* (d), *Glut4* (e), and *adiponectin* (f) in the presence of Igf2 and TNF-α were analyzed in differentiated 3T3-L1 adipocytes treated with TNF-α (3 ng/ml) for 72 h in the presence or absence of Igf2 (400 ng/ml). *P≤0.05, **P≤0.01.

Recent studies suggest that obesity is closely associated with chronic, low-grade inflammation in adipose tissue [14], [15]. Accumulation of adipose tissue macrophages in adipose tissue has been linked to increasing body weight, adipose tissue inflammation and insulin sensitivity. Monocyte chemoattractant protein (MCP)-1, which is expressed in macrophages, is also expressed in adipose tissues. Adipocyte-derived MCP-1 induces macrophage migration into adipose tissue and stimulates the secretion of inflammatory cytokines including TNF-α, which ultimately leads to adipocyte dysfunction [16]. In addition, MCP-1 inhibits insulin-dependent glucose uptake, and MCP-1-deficient mice lack insulin resistance [17]. Adiponectin is also expressed in adipose tissue and modulates several metabolic processes that regulate glucose [18]. In WAT, *MCP-1*expression was much higher, and *Glut4* and *adiponectin* expression was lower in B6 mice fed a HFD than in B6 mice fed a control diet (Fig. 5a, b, c). Therefore we tested whether changes in the expression of *Igf2* and *Peg3* have an effect on adipose tissue inflammation using 3T3-L1 cells. First, we analyzed the effect of Igf2 on the expression of *MCP-1*. Igf2 protein treatment for 72 h decreased *MCP-1* expression in differentiated 3T3-L1 adipocytes (Fig. 5d). Furthermore, low-dose TNF-α (3 ng/ml for 72 h) treatment of differentiated 3T3-L1 adipocytes, in the presence of Igf2, also repressed the expression of *MCP-1* (Fig. 5d). We next examined the effect of Igf2 on insulin signaling in adipocytes. Igf2 treatment up-regulated *Glut4* expression in adipocytes, alone, as well as in the presence of TNF-α (Fig. 5e). In addition, Igf2 treatment up-regulated adiponectin expression in adipocytes (Fig. 5f). These results suggest that Igf2 has an anti-inflammatory effect on TNF-α-induced *MCP-1*, *Glut4*, and *adiponectin* expression in adipocytes.

We also analyzed the effect of down-regulation and up-regulation of *Peg3* on the expression of *MCP-1*, *Glut4* and *adiponectin* in differentiated 3T3-L1 adipocytes. However, the down-regulation or up-regulation of *Peg3* had no effect on the expression of *MCP-1*, *Glut4*, or *adiponectin* (data not shown).

### Igf2 Affects Glucose uptake in Adipocyte

**Figure 6:**
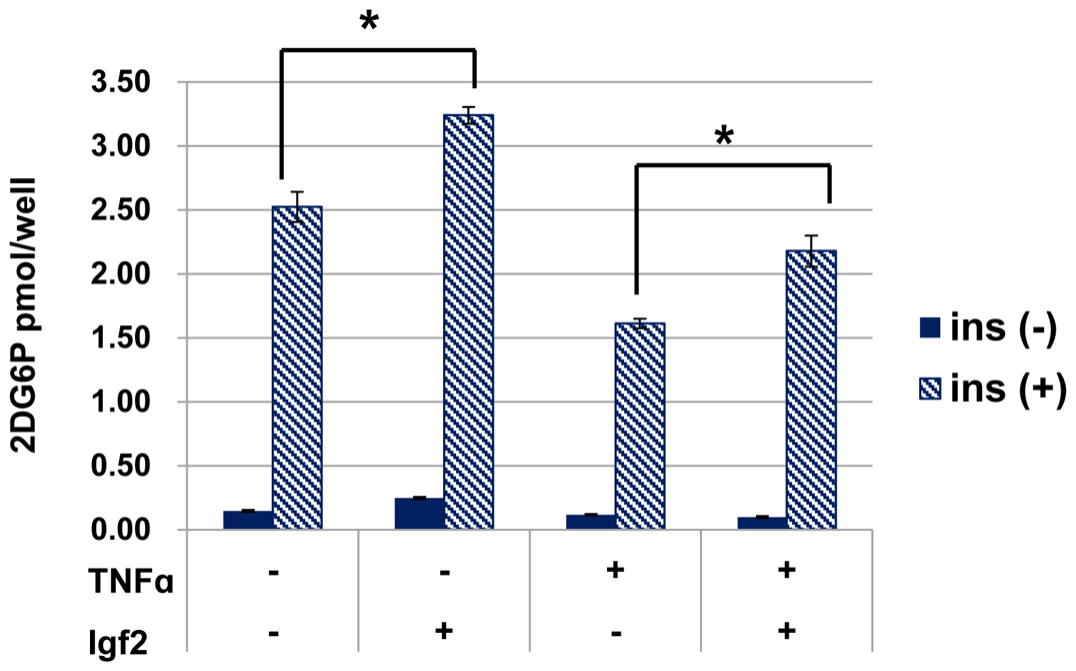
Insulin-stimulated glucose uptake in differentiated 3T3-L1 adipocytes. Differentiated 3T3-L1 adipocytes were treated with or without TNF-α (3 ng/ml) for 72 h in the presence or absence of Igf2 (400 ng/ml). *P≤0.05.

Obesity leads to an increase in TNF-α expression in WAT, which ultimately leads to adipocyte dysfunction [16]. To determine whether Igf2 protects the adipocytes from the physiological effects of TNF-α, we analyzed insulin-stimulated glucose uptake in 3T3-L1 adipocytes in the presence of TNF-α with or without Igf2 treatment. As shown in Fig. 6, Igf2 treatment increased the insulin-stimulated glucose uptake in the presence or absence of TNF-α. These findings imply that down-regulation of Igf2 in adipocytes from HFD-induced obesity mice may result in inflammation and down-regulation of glucose uptake.

### Glut4 Expression in Muscle is Higher in PWK Mice than in B6 Mice

**Figure 7:**
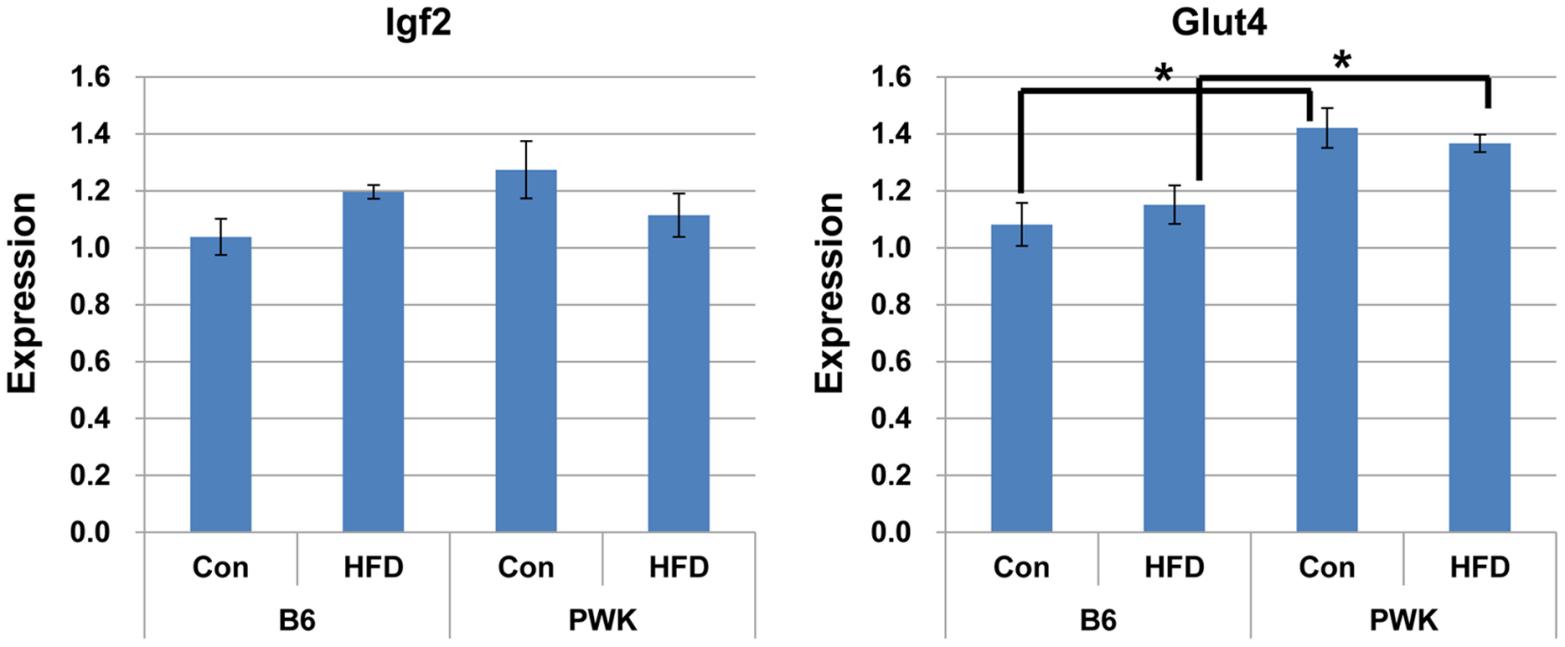
*Igf2* and *Glut4* expression in muscle of B6 and PWK mice fed a control diet or a HFD. *P≤0.05.

Muscle is a major tissue that affects glucose clearance and *Igf2* increases glucose uptake into muscle [19], [20]. We analyzed *Igf2* expression in muscle of B6 and PWK mice fed a control diet or a HFD (Fig. 7). In contrast to WAT, *Igf2* and *Glut4* expression in muscle did not differ between mice fed a control diet and mice fed a HFD. However, *Glut4* expression in muscle was higher in PWK mice than in B6 mice.

## Discussion

In this study, we report evidence for paternal transmission of HFD-induced obesity, which correlated with the expression of *Peg3* and *Igf2* imprinted genes. We found that PWK mice are resistant to HFD-induced obesity compared to C57BL/6J (B6) mice. Using the reciprocal crosses between these mice, we found that (PWK×B6) F1 mice are more sensitive to dietary obesity compared to (B6×PWK) F1 mice, suggesting a paternal transmission of diet-induced obesity. One way by which paternal transmission might occur is genomic imprinting.

Our findings suggest that down-regulation of *Peg3* and *Igf2* in adipocytes contributes to diet-induced obesity and the symptoms associated with obesity. Several imprinted genes are associated with body weight dysregulation, and we found that *Peg3* and *Igf2* might be involved in the paternal transmission of propensity to diet-induced obesity. *Peg3* is a paternally expressed gene and previous reports have shown that targeted mutation of *Peg3* results in increased body fat in mice [7]. In addition, *Peg3* is a candidate gene for the regulation of intramuscular fat content in pigs [21], suggesting its involvement in adipocyte differentiation. *Igf2* is also a paternally expressed gene and *Igf2*-deficient mice are severely growth retarded [22]. *Igf2* acts as part of the IGF signaling pathway to regulate postnatal growth, and decreased expression of *Igf2* is associated with increased fat mass in a mouse model [23]. In adult humans, lower circulating IGF2 levels have been associated with an increased risk of weight gain and obesity [24]. In addition, several studies have reported the correlation between *Igf2* genotype and obesity in humans [25], [26].

In addition to fat gain, *Peg3* and *Igf2* might also contribute to the symptoms associated with obesity. Chronic low-grade inflammation in adipose tissue is an important feature of obesity and type 2 diabetes. In WAT, *MCP-1*expression was much higher, and *Glut4* and *adiponectin* expression was lower in B6 mice fed a HFD than in B6 mice fed a control diet.MCP-1 stimulates the secretion of inflammatory cytokines including TNF-α, and ultimately leads to adipocyte dysfunction. Adiponectin is an anti-inflammatory cytokine and enhances insulin action by reducing ectopic fat deposition [27], and a low level of circulating adiponectin is closely associated with type2 diabetes [28]. We therefore analyzed the effect of expression changes of the paternal imprinted genes, *Peg3* and *Igf2*, in in vitro studies using differentiated 3T3-L1 cells. We demonstrated that Igf2 effectively inhibited TNF-α-induced increase of *MCP-1* expression in 3T3-L1 adipocytes and up-regulated *Glut4* and *adiponectin* transcription. In addition, we also showed that Igf2 increased the insulin-stimulated glucose uptake in 3T3-L1 adipocytes together with a low-dose TNF-α treatment. These results suggest that Igf2 has an anti-inflammatory effect and enhances glucose metabolism. However, we did not detect an effect of *Peg3* down-regulation or up-regulation on the expression of *MCP-1*, *Glut4*, or *adiponectin*. Further studies are needed to understand the effect of *Peg3* on chronic low-grade inflammation.

The glucose tolerances of B6 and PWK mice fed a control diet drastically differed. In B6 mice, a HFD is associated with glucose tolerance due to changes in insulin sensitivity and insulin secretion [29], [30], [31], [32]. The difference in glucose clearance between B6 and PWK mice might be due to a difference in insulin secretion or insulin sensitivity. One possible factor is the serum adipokine concentration. Adipose tissue produces and secretes adipokines such as adiponectin [33], and adiponectin has insulin-sensitizing and glucose-clearing effects [34], [35]. A high serum concentration of adiponectin might contribute to the enhanced glucose clearance and insulin sensitivity of PWK mice compared to B6 mice. *Glut4* expression in muscle was higher in PWK mice than in B6 mice, suggesting that the efficiency of glucose uptake by muscle was higher in PWK mice than in B6 mice. This could also explain the difference in glucose clearance between PWK and B6 mice. Additional studies are required to determine the mechanism underlying the enhanced glucose clearance of PWK mice compared to B6 mice.

Down-regulation *of Peg3* and *Igf2* in adipocytes could contribute to diet-induced obesity and the symptoms associated with obesity. However, our findings raise an interesting question of why *Peg3* and *Igf2* are down-regulated in the adipose tissue of HFD-induced obese B6 mice and not in HFD-induced obese PWK mice, which will be the focus of future studies in our laboratory.

Most imprinted genes have one or more regions in which the maternal or the paternal alleles are differentially methylated, and the DNA methylation states are associated with the expression of imprinted genes. *Peg3* and *Igf2*, like other imprinted genes, are regulated by DMR. We found that *Peg3* and *Igf2* are down-regulated in adipocytes of diet-induced obese B6 mice compared to control mice. However, the DNA methylation of the imprinting control region of *Peg3* and *Igf2* was not changed, indicating that DNA methylation is not involved in the expression changes of these genes. We are now investigating the mechanism of down-regulation of *Peg3* and *Igf2* in obese B6 mice. Understanding this mechanism will facilitate the development of novel approaches to the treatment of obesity.

The expression of other imprinted genes, *Zac1* and *Grb10,* was significantly reduced in WAT of diet-induced obese B6 and PWK mice. Expression of *Zac1*in WAT was much higher in PWK mice than in B6 mice. *Zac1* is a paternally expressed gene that up-regulates *Igf2* expression through binding to a shared enhancer [36]. The mechanism underlying down-regulation of *Zac1* in WAT of diet-induced obese B6 and PWK mice is unclear; however, the difference in *Zac1* expression between B6 and PWK mice might affect *Igf2* expression.

*Grb10* is a maternally expressed imprinted gene and can interact with a variety of receptor tyrosine kinases, including the insulin receptor [37]. The functional role of Grb10 in insulin signaling is controversial. Some experiments have indicated that Grb10 is a positive regulator of insulin signaling, while others have indicated that Grb10 inhibits insulin action [37]. In addition, mice in which *Grb10* is disrupted exhibit reduced adiposity [38]. The mechanisms underlying down-regulation of *Grb10* in WAT of diet-induced obese B6 and PWK mice, and the higher expression of *Grb10* in B6 mice fed a control diet than in PWK mice fed a control diet are unknown. Expression of *Dlk1*, which is a paternally expressed imprinted gene, varied widely in WAT of B6 and PWK mice; therefore, there was no statistically significant difference between the strains.

In conclusion, we report that HFD-induced obesity is paternally transmitted and is correlated with expression of the *Peg3* and *Igf*2 imprinted genes, and that down-regulation of *Peg3* and *Igf2* in adipocytes can contribute to diet-induced obesity and the symptoms associated with obesity. These findings suggest that *Peg3* and *Igf*2 improve obesity and glucose metabolosm in mice fed a HFD.
